# Microbial monitoring and methods of sample collection: a GITMO survey (Gruppo Trapianto di Midollo Osseo)

**DOI:** 10.3332/ecancer.2014.421

**Published:** 2014-04-10

**Authors:** Erica Gori, Emanuela Callea, Francesca Alberani, Laura Orlando

**Affiliations:** 1 Institute of Haematology and Medical Oncology ‘L&A Seragnòli’, Bologna, Italy; 2 Hospital Hygiene Service, Bologna Hospital, Bologna, Italy; 3 European Institute of Oncology, Milan, Italy

**Keywords:** microbial monitoring, sample collection

## Abstract

The collection of microbiological samples represents an important aspect of care both for doctors as well as nurses. It is important to recognise and identify some key points, to avoid performing ‘unnecessary’ or ‘incorrect’ sampling, which may give useless or even misleading results, these are: the moment at which the sample is collected, the collection method and timing (if indicated). The comparison between the various nursing members of the Italian National bone marrow transplant group (GITMO), showed diversity of practice across all fields. A formal survey was therefore conducted within GITMO centres looking at the methods of microbiological sample collection. These results were compared with the literature, and in addition to the lack of homogeneity of practice within the centres, a lack of compliance with the recommendations was also observed. To evaluate the effectiveness of this survey in highlighting awareness of this issue and the presence of relevant guidelines, the questionnaire was repeated (with the same centres responding), which demonstrated no major changes in care practices.

**Conclusion:**

The survey has allowed us to highlight many critical issues regarding common procedures which are not commonly discussed. Considerable differences were noted between different transplant centres, which may be attributable to the lack of Italian guidelines that can be used as a starting point for clinical practice. The plenary discussion allowed for an exchange of findings with the medical staff, who are usually responsible for requesting microbiological samples. The ideal solution would be a unique field-based training programme, associated with the dissemination of a common procedural document for ensuring evidence-based practice.

## Introduction

**Figure 1:**
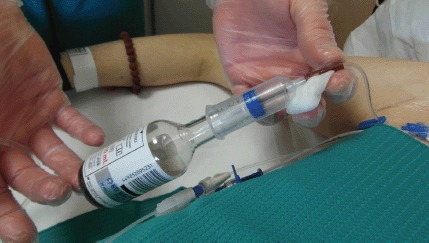
Procedure for blood sampling.

The collection of microbiological samples (blood cultures, urine cultures, and swabs), represents an important moment, not only for haematological patients, but for all types of patients. We know from the literature that blood cultures performed incorrectly can give false positive results, which results in the misuse of antibiotics and the use of further diagnostic investigations, resulting in increased costs, longer hospital stays, and potential antibiotic resistance. We also know that careful attention must be paid to the collection of the blood culture (Figure 1) because contamination can occur through various sources (the environment, the operator’s hands, the devices used to fill the bottles, the patient’s skin, and the material used to perform the blood sample).

At the nursing session of the Italian National Bone Marrow Transplant Meeting (GITMO) in 2009, during a debate surrounding the collection of microbiological samples, numerous differences were observed in the timing and method of collection. The nurse lead for infection within the GITMO nursing group was given the responsibility to provide responses to the doubts highlighted, with the ultimate aim of providing universal indications, to standardise practice.

The work was undertaken in five distinct phases, the first being an analysis of the existing literature to identify that pertinent to current practice and this clinical setting; the second phase, run jointly with the GITMO Board of Directors, was to design and send out a questionnaire to collect information regarding the methods of microbiological sample collection; the third phase was to analyse results by means of a database; the fourth phase was to compare results with the literature identified in phase I and, finally, to disseminate the findings of the survey to colleagues.

The completed work was to be presented at the 2010 GITMO National Meeting, allowing for both nursing and medical comments on findings. During this occasion, given the importance of the results, the need to share findings with the medical team was evident. Therefore, the GITMO Board requested that the nursing lead for ‘infections’ within the GITMO nursing group was again given the responsibility to re-send the questionnaire, with slight modifications, eight months after the presentation of the results to see if, over the course of the previous months, any changes had been made to clinical practice in order to comply with the evidence that had been presented. 

## Objective

The primary objective was to apply evidence-based practice (EBP) in response to the doubts raised, and using evidence from the literature, improve healthcare practice.

The secondary objectives were:

- to standardise practice within GITMO Italian transplant centres;
- to verify the daily practices of the transplant centres, analysing the issued identified;
- to share these findings with nursing and medical staff, to critically analyse current practice;
- to ensure that the findings and data are available on the GITMO website for open consultation.

## Methods

### Phase 1: Literature review

A literature review was performed of the main international databases: PubMed, Centres for Disease Control and Prevention (www.cdc.gov), Infectious Diseases Society of America guidelines, NGC (National Guideline Clearinghouse), Royal College of Nursing, and the Cochrane Library. Among these articles, guidelines have been selected, with particular attention to recent publications, source of the document and recommendations. Five key documents were highlighted:

- Taking blood cultures: a summary of best practice (National Health Service) [1].
- Clinical and Laboratory Standards Institute. Principles and Procedures for Blood Cultures; Approved Guideline (Clinical and Laboratory Standards Institute) [2].
- Blood Cultures in the Critical Care Unit (Shafazand and Weinacker) [3].
- Infectious Diseases Society of America. Guideline for Intravascular Catheter-Related Infection (Clinical Infections Diseases) [4].
- Clinical Practice Guideline for the use of antimicrobial agents in neutropenic patients with cancer: 2010 update by the Infectious Diseases Society of America (Infectious Diseases Society of America) [5].

### Phase 2: Questionnaire development and distribution

A questionnaire was developed, consisting of 16 multiple-choice questions. It was divided into five sections: the first in which transplant centre details where general characteristics of the centre were recorded, and the remaining four sections with questions regarding methodology and timing of blood culture collections, swabs, stool cultures, and urine cultures.

The questionnaire was piloted amongst members of the GITMO Board, to test the clarity and validity of the questionnaire in its measurement of the issues. Once validated, the questionnaire was sent in January 2010 via email to all 88 GITMO transplant centres. Of these, 48 questionnaires were completed correctly and submitted for analysis.

The questionnaire was sent out for a second time after the presentation of the findings at the GITMO National Meeting in January 2011. This time, the questionnaire was sent to the 48 centres that completed the questionnaire in the first wave in 2010. With this questionnaire, the original responses from that centre were sent as an attachment, asking responders to check whether these were still correct, or if any current practices had been changed during the time following the first questionnaire. Where there had been changes in practice, responders were asked to give reasons why the change was implemented. 

### Phase 3: Data analysis

Data from both the 2010 and 2011 questionnaires were collected in Excel spreadsheets, and a descriptive analysis was performed. The analysis of the 2011 data compared responses between 2010 and 2011, to highlight any differences.

#### Data analysis (results of the first questionnaire in 2010)

The questionnaires were sent to 88 centres of which 48 were completed correctly, and therefore could be analysed. Of the 48 centres responding, 29 were adult, eight paediatric, and 11 mixed (adult and paediatric) transplant centres. Of these, all centres were involved in chemotherapy administration, and in addition, 30 centres performed both autologous and allogeneic hematopoietic stem cell transplants (HSCT) and 18 centres performed only autologous HSCT.

Regarding the questionnaire, the first 12 questions related to the methods of blood culture collection, a summary of which is provided. The questionnaire provided multiple-choice options and also the possibility to report other practices not specified in the lists provided.

Statement: ‘Blood cultures are performed…’Thirty-seven centres (77%) reported performing blood cultures when patients were febrile >38 °C with or without rigors. Twenty-six centres (54%) performed cultures at the first temperature spiked >38 °C after HSCT, 19 centres (39%) performed blood cultures when patients experienced rigors, even in the absence of fever. Sixteen centres (33%) took blood cultures at the time of admission into the inpatient unit, two centres (4%) performed sampling every week following hospital admission, and a further four centres (8%) reported ‘other’ clinical practice regimens (e.g., at patient admission if the patient had a central venous catheter (CVC)).

The literature indicates that ‘*blood cultures should only be taken when there is a clinical need to do so and not as routine. Blood cultures should be taken after the identification of possible bacteraemia or sepsis and before the administration of antibiotics. If a patient is on antibiotics, blood cultures ideally should be taken immediately before the next dose, with the exception of paediatric patients. Blood cultures should only be collected by members of staff (medical or nursing) who have been trained in the collection procedure and whose competence in blood culture collection has been assessed and documented*’ (Taking blood cultures. A summary of best practice—NHS [1]).

*Statement: ‘In neutropenic patients, after the first temperature spike and start of antibiotic therapy, blood cultures are repeated…’ * Twenty-two centres (46%) repeated cultures at every episode of fever >38 °C with or without rigors, 13 centres (27%) repeated cultures every time rigors were present—even where patients were apyrexial and 13 centres (27%) did not repeat any blood cultures within the first 72 h after the start of empirical antibiotic therapy. Fifteen centres (31%) repeated blood cultures serially according to internal guidelines, six centres (12%) responded ‘other’ (e.g., blood cultures 48 h after the first set of cultures, once daily and so on).

The literature indicates that ‘*blood cultures should not be repeated for 2–5 days. The use of so-called surveillance blood cultures has been advocated as a means to allow earlier detection of sepsis in certain patient populations (…such as those undergoing transplantation or with vascular catheters)…however this should not be performed routinely, as these cultures do not improve patient management but add substantial costs’ *[6–8]—cited in CLSI. Principles and Procedures for Blood Cultures; Approved Guideline. Clinical and Laboratory Standards Institute [2].

*Question: ‘From which sites are blood culture samples taken…’ *It should be pointed out that patients undergoing HSCT always have a central venous access device (CVAD). That said, 36 centres (75%) take samples both via the CVAD and peripheral blood; ten centres (21%) take samples only from the CVAD, and two centres (4%) perform sampling only from peripheral blood. It was interesting to note that of the ten centres that take samples only from the CVAD, five were paediatric, three were mixed, and two were adult centres.

The literature indicates that ‘*Peripheral venepuncture is the preferred method for blood sampling. Blood culture samples drawn from intravascular catheters are not optimal, as they can become contaminated with organisms colonizing the hub or the walls of the catheter. If it becomes necessary to obtain culture samples from these catheters, strict aseptic technique should be followed, while all efforts are made to draw a second set of culture samples from a peripheral venepuncture’—*Blood Cultures in the Critical Care Unit: Improving Utilization and Yield—Shafazand [3].

‘*In neutropenic oncology patients, during the initial assessment of fever, it is recommended that at least 2 sets of blood cultures are performed, with a set collected simultaneously from each lumen of an existing central venous catheter (CVC), if present, and from a peripheral vein site; 2 blood culture sets from separate venepunctures should be sent if no central catheter is present. Some experts suggest taking samples only from CVAD’s, without peripheral blood sampling, however the expert panel does not support this approach to the initial assessment, as catheter related blood stream infection (CRBSI) cannot be ruled out without a simultaneous blood sample from a peripheral vein*.’ Clinical Practice Guideline for the use ofAntimicrobialAgents in Neutropenic Patients with cancer: 2010 Update by the Infectious Disease Society of America—IDSA GUIDELINE [5].

‘*Blood cultures taken from central catheters are associated with a greater risk of contamination compared with those obtained from a peripheral device. Blood cultures from central catheters should be performed along with peripheral blood culture, in order to allow a better interpretation of the results in case of positivity.’*—CLSI. Principles and Procedures for Blood Cultures; Approved Guideline. Clinical and Laboratory Standards Institute [2].

*Question ‘To bleed or not to bleed ?’ *By bleeding, we are referring to the first few ml of blood taken and whether this should be discarded before executing the blood culture. In general, for routine biochemical tests, the first few ml is discarded to avoid the presence of heparin or medication residue affecting the reliability of the results. Form the questionnaire, 39 centres (81%) reported that they did not discard any blood when taking blood cultures, whilst nine centres (19%) discarded the first few ml.

The literature indicates that ‘*if the blood culture is collected through an intravenous line, it is not necessary to discard the initial volume of blood or flush the line with saline to eliminate residual heparin or other anticoagulants *[9]*. Moreover, the antimicrobial activity of heparin is eliminated in protein-rich culture media *[10, 11].’—CLSI. Principles and Procedures for Blood Cultures; Approved Guideline. Clinical and Laboratory Standards Institute [2].

*Statement: ‘Sterile gloves are worn when…’ *Twenty-four centres (50%) reported wearing sterile gloves during blood culture sampling from both CVAD’s and peripheral venous devices, 12 centres (25%) wear gloves only where it is necessary to re-palpate the skin after being disinfected in peripheral blood culture sampling. Nine centres (19%) reported wearing gloves only for CVAD blood culture sampling, two centres (4%) only wear gloves for peripheral blood culture sampling, two centres (4%) reported never wearing gloves, and two centres did not respond.

The literature suggests that ‘*the person drawing the culture should not palpate the vein after skin disinfection unless a sterile glove is worn*.’*—*CLSI. Principles and Procedures for Blood Cultures; Approved Guideline. Clinical and Laboratory Standards Institute [2].

‘*To avoid cross contamination from the hands of the person drawing the culture, it is fundamental not to re-palpate the site after disinfection*.’*— *Taking blood cultures. A summary of best practice—NHS [1].

*Question: ‘Which disinfectant is used for cleansing prior to peripheral venepuncture…’ *Thirty-one centres (64%) used chlorhexidine, 16 centres (33%) povidone–iodine, two centres (4%) used iodine compresses, three centres (6%) did not respond, and one centre reported that chlorhexidine was no longer available.

The literature suggests that ‘*Skin preparation for obtaining percutaneously drawn blood samples should be performed carefully, with use of either alcohol or tincture of iodine or alcoholic chlorhexidine (>0.5%), rather than povidone–iodine; allow adequate skin contact and drying times to mitigate blood culture contamination.*’—IDSA Guideline for Intravascular Catheter-Related Infection*—*CID [4].

*‘Alcoholic solution products were better in the reduction of BCC (blood culture contamination) rate. Between alcoholic chlorhexidine and povidone–iodine, the first should be preferred. Chlorhexidine was demonstrated to be well tolerated, whereas iodine products can be skin irritants *[12, 13]*. Alcoholic chlorhexidine needs 15 to 30 s to dry; iodine tincture dries in 30 s, whereas povidone–iodine needs about 2 min *[14–17]*. For convenience, alcoholic chlorhexidine appears to be the best antiseptic for medical staff, as they need to maximise efficiency and resources in the shortest time possible. Alcoholic chlorhexidine solutions showed statistically significant reduction in blood culture false-positives compared with aqueous povidone–iodine.’*—Skin antiseptic in venous puncture-site disinfection for prevention of blood culture contamination: systematic review with meta-analysis—Journal of Hospital Infection—Caldeira *et al *[18].

*Statement: ‘Degreasing of the skin with alcohol before disinfection, in the case of peripheral venepuncture…’ *Forty-four centres (92%) did not perform degreasing, only two centres (4%) undertook this practice, and two centres (4%) did not respond.

The literature suggests that ‘*the site of the venepuncture should be disinfected; typically this means cleansing the site first with 70% isopropyl alcohol (in Italy 70% alcohol is used as isopropyl alcohol is not available) allowing it to air dry, following the application of the main disinfectant, then allowing that substance to sit for the recommended amount of time.*’—CLSI. Principles and Procedures for Blood Cultures; Approved Guideline. Clinical and Laboratory Standards Institute [2].

*Statement: ‘The number of pairs of blood culture bottles taken in the case of multi lumen CVAD’s (by pairs it is intended the set of bottles for aerobic and anaerobic blood culture sampling)…’ *Twenty-five centres (52%) reported taking one pair of blood cultures from each lumen of the CVAD, 17 centres (25%) reported taking only one pair of cultures, one centre (2%) did not respond, and five centres responded ‘other’ (e.g., pairs from only one lumen, pairs from only two lumens and so on).

The literature suggests that ‘*the volume of blood drawn for culture is the most important variable in detecting bacteraemia or fungemia *[19–28]*. This observation is based on data published many studies of adult patients with bacteraemia or fungemia. For adult patients, the recommended volumes for blood cultures are 20–30 ml per culture (i.e., per venepuncture). For infants and younger children, the volume of blood drawn should be no more that 1% of the patients total blood volume.’—*CLSI. Principles and Procedures for Blood Cultures; Approved Guideline. Clinical and Laboratory Standards Institute [2].

‘*The total volume of blood cultured is a crucial determinant of detecting a blood stream infection *[23]*. At least 2 sets of blood culture specimens should be obtained, (a ‘set’ consists of 1 venepuncture or catheter access draw of ~20 mL of blood divided into 1 aerobic and 1 anaerobic blood culture bottle). In paediatric patients weighing less than 40 kg, the volume of blood drawn should be no more that 1% of the patients total blood volume*.’*—*Clinical Practice Guideline for the use of Antimicrobial Agents in Neutropenic Patients with cancer: 2010 Update by the Infectious Disease Society of America—IDSA GUIDELINE [5].

*‘A “set” consists of 1 venepuncture or catheter access draw of ~20 mL of blood divided into 1 aerobic and 1 anaerobic blood culture bottle.’—*Clinical Practice Guideline for the use ofAntimicrobialAgents in Neutropenic Patients with cancer: 2010 Update by the Infectious Disease Society of America—IDSA GUIDELINE [5].

*Question ‘Blood culture test for real time PCR (SEPTIFAST)…’ *37 centres (77%) did not perform this test, eight centres (17%) performed this test, and three centres (6%) did not respond.

‘SeptiFast rapidly diagnosed blood stream infections in a cohort of immunosuppressed patients, and it is suggested that SeptiFast can be used in conjunction with, but cannot replace, blood cultures to better identify the etiology of fever in immune-compromised patients’ [29].

*Statement: ‘Tests performed on routine surveillance blood culture samples…’ *Thirty two centres (67%) tested for aerobes, anaerobes and fungi, 12 centres (25%) tested for aerobes and anaerobes, three centres (6%) responded ‘other’ (e.g., only aerobes, aerobes, and fungi), and one centre did not respond.

‘*Because the data are conflicting and inconclusive, and because the recommendation that anaerobic blood culture bottles be limited to use in select patient populations has never been validated by controller clinical studies, it is recommended that routine blood cultures include paired aerobic/anaerobic blood culture bottles, it is recommended that routine blood cultures include paired aerobic/anaerobic blood culture bottles *[30–32]*. When less than the recommended volume of blood is drawn for culture, the blood should be inoculated into the aerobic vial first.*’*—*CLSI. Principles and Procedures for Blood Cultures; Approved Guideline—Clinical and Laboratory Standards Institute [2]. 

*Statement: ‘Needleless systems, if used, are removed before the blood sample is taken…’ *Twenty-four centres (50%) did not remove the system before taking the blood culture sample, 16 centres (33%) removed the needleless system, two centres (4%) did not use needleless systems, and six centres (12%) did not respond.

The IDSA Guidelines of 2009 [4], speak about cleansing of the catheter hub when sampling from a CVAD. Given the scarcity of reference material on the use of needleless devices, and of the variety of approaches within the nursing group, (some claiming that the blood sample should be taken with the device *in situ*, others claiming it necessary to remove the device to avoid false positive results), the IDSA were contacted directly. The IDSA expert forwarded the one article available [33], in which an increased incidence of false-positives was reported in cases where blood culture withdrawals were made with the needleless connector in place. Therefore, the indication (although lacking strong recommendations) is that of replacing the needleless connector prior to sampling or taking the sample directly from the CVAD hub.

*Question: ‘Is there an interval between blood culture sampling?’ *The centres in this case were perfectly divided, 50% (24 centres) taking samples at separate times, and 50% taking the samples at the same time.

Research suggests that ‘*the ideal interval between blood cultures is not well known, but likely has less impact on the yield than was once thought *[19]*. In a study of the optimal time interval between blood cultures, Li et al *[19] *demonstrated that similar yields were obtained when samples were collected simultaneously, within 2 h, or within 24 h of the initial blood culture. In critically, ill patients who are heamodynamically unstable, two blood culture sets should be drawn promptly prior to initiation of empiric antibiotic treatment.’ —*Blood Cultures in the Critical Care Unit, Shafazand and Weinacker [3].

*‘Only a few studies have tried to establish the optimal timing of blood cultures to maximise the recovery of pathogens from blood. Although it has been common practice to obtain blood cultures at arbitrary intervals of 30 to 60 min *[34]*, Li et al *[19] *showed no difference in microbial recovery when blood specimens were drawn for culture simultaneously or at spaced intervals for up to 24 h. Blood cultures should be obtained simultaneously (or over a short time frame). Drawing blood at intervals is only indicated when it is necessary to document continuous bacteraemia in patients with suspected infective endocarditics or other endovascular (e.g., catheter related) infections.’—*CLSI. Principles and Procedures for Blood Cultures; Approved Guideline—Clinical and Laboratory Standards Institute [2].

The three final questions were to investigate the timing of collection of urine and stool cultures and microbiological swabs.

*Statement: ‘Taking microbiological swabs…’ *Thirty-six centres (75%) take swabs only as required, 31 centres (64%) perform swabs as routine at hospital admission, 13 centres (27%) perform routine swabs weekly from hospital admission, eight centres (17%) take swabs after the first spike of fever after HSCT, one centre (2%) took swabs weekly after the first spike of fever, one centre took weekly swabs after HSCT, and five centres (10%) reported ‘other.’

*Statement: ‘Taking urine cultures…’ *Forty two centres (87%) performed urine culture samples only in cases where patients reported clinical symptoms or cystitis, 21 centres (44%) performed urine culture at admission, ten centres (21%) performed weekly urine cultures from hospital admission, ten centres (21%) performed urine cultures only in patients with urinary catheters, six centres (12%) took urine cultures following the first temperature spike following HSCT, three centres (6%) performed urine cultures weekly after HSCT, two centres (4%) performed weekly cultures after the first temperature spike, and six centres responded ‘other.’

*Statement: ‘Stool cultures are taken…’ *Forty two centres (87%) took stool culture samples only in cases of diarrhoea, 14 centres (29%) took samples at hospital admission, five centres (10%) took weekly samples from the time of hospital admission, one centre perform culture samples weekly following HSCT, two centres (4%) perform culture samples after the first temperature spike after HSCT, and five centres (10%) responded ‘other.’

The literature suggests that ‘*HSCT centre personnel should not perform routine fungal or bacterial cultures of asymptomatic HSCT recipients*’ [35, 36].

‘*Culture of sites should be guided by clinical signs and symptoms but should not be performed routinely.*’*—*Clinical Practice Guideline for the use of Antimicrobial Agents in Neutropenic Patients with cancer: 2010 Update by the Infectious Disease Society of America—IDSA GUIDELINE [5].

#### Data analysis Part II (data from the 2011 survey)

Given the importance of this topic, eight months later, it was decided to test the effectiveness of the educational work undertaken by re-submitting the questionnaire to each of the 48 centres that initially responded correctly to the questionnaire. An allocated nurse within each centre was identified and asked to verify if the responses given previously were still valid or where practice had changed, to identify their new practice regarding microbiological sampling. The questionnaire included preliminary questions about changes that had occurred.

Of the 48 centres, only 38 completed the questionnaire correctly. The 38 transplant centres were: 24 adult centres, six paediatric centres, and eight mixed centres (paediatric and adult). Of these, 13 centres performed both autologous and allogeneic HSCT and 25 centres performed autologous HSCT and chemotherapy regimens.

The centres were asked, after the presentation of the initial results and the literature surrounding microbiological sampling at the GITMO National Meeting in 2010, whether anything had changed in the internal procedures, of which 11 centres responded ‘yes’, 26 centres responded ‘no’, and one centre did not respond. Centres were then given the opportunity to justify their response, with a series of multiple-choice questions within the questionnaire.

Amongst the centres reporting changes in their procedures, only four attributed this to the educational value of the work at the National Meeting, the other ten centres reported that changes were made to comply with internal regulations.

Amongst the centres that reported no changes in their practice, 24 centres reported that their practice was already in line with the work presented at the National Meeting. Four centres did not respond and one centre responded ‘other.’

The analysis of the 2011 data were found to be almost the same as that of 2010, with minimal differences relating to the timing of blood culture sampling and use of sterile gloves.

### Phase 4: Comparing results with the literature

The data obtained was compared with the current literature in this field, highlighting critical issues, with the aim of following the principles of EBP, correcting non-evidence-based practices.

The differences between the guidance from the literature and practice findings, are probably due to a variety of factors which may include the lack of Italian documentation/ literature that may be used as a starting point for EBP, the absence of evidence-based nursing (EBN) centres within many of the transplant centres to act collaboratively with the Infection Control Department and basically, the lack of an Italian guideline according to EBP criteria.

Analysing the data, it is possible to note some practices that appear devoid of rationale, but are so deeply rooted over the years, that they are difficult to change.

## Discussion

The literature tells us that bacterial or fungal cultures should not be performed in asymptomatic patients and that cultures from sites of suspected infection should be performed only if clinically indicated, and not as routine.

The analysis of the data, however, shows a more or less widespread tendency to perform certain types of routine microbiological tests.

Perhaps this is due to the lack of reference documents in Italian, or internal procedures that are difficult to change. Another factor is possibly the limited number of EBN centres that work actively within clinical centres, teaching staff how to apply the rules of EBP.

The literature always emphasises the diagnostic importance of blood cultures, and hence the importance of the manner in which the sample is collected. Contamination of blood cultures can come from several sources: the material used, the patient’s skin and the hands of the person taking the sample. Another decisive factor is that of the amount of blood collected. The lack of accuracy in performing blood culture sampling, can lead to false positive results, which may lead to unnecessary antibiotic therapy and an increase in hospital stay and costs, and not least, an increased antibiotic resistance.

Shafazand & Weinacker [3]: ‘The judicious use of cultures will improve the utility of blood cultures as diagnostic tools in critically ill patients.’

### Phase 5: Dissemination of results and discussion with colleagues

The results obtained and the corresponding literature were presented at the Italian National Transplant meeting (GITMO) in 2011 in a plenary session, and are available on the GITMO website.

## Conclusion

This survey has allowed us to highlight many criticisms of procedures that are widely used, yet have been little discussed. Considerable differences have been noted between the various transplant centres, certainly attributable to the lack of official Italian documentation as a basis for practice.

The plenary discussion allowed for an exchange of findings with the medical staff, who are usually responsible for requesting microbiological samples.

Over the eight month period from completion of one questionnaire to another, changes in practice were few and insignificant. In order to obtain positive results in terms of evidence-based practice implementation, it would probably be necessary to have a field-based training programme, in association with the dissemination of a common procedural document for ensuring EBP.
